# Association between bruxism severity and serum concentrations of 25‐hydroxyvitamin D levels

**DOI:** 10.1002/cre2.530

**Published:** 2022-02-06

**Authors:** Bushra A. W. Allaf, Mahmoud Abdul‐Hak

**Affiliations:** ^1^ Department of Oral Medicine Faculty of Dentistry, University of Damascus Damascus Syria; ^2^ Department of Oral Medicine Faculty of Dentistry, Arab International University Ghabaghib, Daraa Governorate Damascus Syria

**Keywords:** 25‐hydroxyvitamin D, bruxism, concentrations, serum, severity

## Abstract

**Objectives:**

The aim of this study was to investigate the possible relationship between bruxism and blood levels of 25‐hydroxyvitamin D.

**Material and Methods:**

One hundred male and female patients from Damascus joined the study. Their ages were in the range of 18–40 years. Two groups were considered in this study, the first group included patients who were already suffering from bruxism (76 patients) and the second group consisted of patients who were not suffering from any bruxism (24 patients). The analysis of the blood samples for 25‐hydroxyvitamin D levels of the two groups was carried out.

**Results:**

The recorded results showed that there is a relationship between the levels of bruxism and 25‐hydroxyvitamin D concentration in the blood (*p* < .05), enhancing that the bruxism activity has been shown the associated deficiency in 25‐hydroxyvitamin D levels, which can be divided into five different categories.

**Conclusions:**

Within the limitations of this study in spite of the number of patients (one hundred), according to the reported study, it appears that there is a significant relationship between bruxism and the level of 25‐hydroxyvitamin D in the blood.

## 1 BACKGROUND

Bruxism is defined as a parafunctional activity of the masticatory muscles, which is characterized by the unconscious clenching and/or grinding of the teeth, and it may occur at any time of the day (Veiga et al., 2015). This activity has a multifactorial etiology associated with psychological factors, local factors, personal characteristics, and also disruption of sleep (Demjaha et al., 2019). Over the past two decades, several studies have highlighted the existence of a central administration in the mechanism of bruxism (Kato et al., 2003; Lavigne et al., 2007). On the other hand, this neurological mechanism is associated with sleep quality and the psychological state (depression, stress, and anxiety) of the individual (Herrero Babiloni & Lavigne 2018). Accordingly, bruxism as an oral condition has great importance to both researchers and doctors in the fields of dentistry, neurology, and sleep medicine (Lobbezoo et al., 2013) and it is not only limited to the state of clenching and grinding of the teeth.

The potential role of 25‐hydroxyvitamin D (25(OH)D) levels, in a wide range of health outcomes has increased (Moon et al., 2017). Vitamin D interferes with the neurological mechanism properties due to the spread of vitamin D receptors (VDR) in multiple areas of the central nervous system (Koundourakis et al., 2016) and also due to its potential role in the decline or improvement in mood and sleep stability of the individuals (Casseb et al., 2019; Gominak & Stumpf, 2012).

Therefore, many studies have shown the associations between low serum 25(OH)D concentrations (which reflects the patient's general vitamin D status) and health, for example: The increased risk of cancer, cardiovascular disease, glucose metabolism disorders, neurodegenerative diseases, and death (Chu et al., 2010).

In addition to that, vitamin D has a function in neuroimmune modulation, neuroplasticity, and reducing neurological oxidative stress (Berridge, 2016) and the relationship between oxidative stress and inflammation to the pathophysiology of psychological disorders which has also recently been reported (Hassan et al., 2014).

A recent review highlighted that compounds that are able to reduce oxidative stress and neuroinflammation may be promising candidates in the treatment of anxiety. Thus, vitamin D, with its antioxidant and anti‐inflammatory properties, stands out as a candidate for managing anxiety disorders as well (Santos et al., 2019). Therefore, an interest to manage the vitamin D blood concentrations and follow its influence on the psychological status of the individuals meets large consideration.

Additionally, some researchers hypothesize that vitamin D has an important function in brainstem control during sleep because many nuclei in the hypothalamus and brainstem (which are known to interfere with sleep) contain high concentrations of vitamin D receptors. Hence, the epidemiology of vitamin D deficiency might be the current epidemiology of sleep disorders (Gominak & Stumpf, 2012).

In this regard, this study is conducted to investigate the possible association that might link between different serum concentrations of 25 (OH)D and the severity of bruxism in otherwise non‐bruxer individuals; this investigation enhances the possibility of finding a relationship that may combine two epidemiological conditions related to psychological and sleep disruption. To the best of our knowledge, no study has yet investigated the association between bruxism and 25(OH)D levels in the blood. This paper investigates this association for a total of 100 Syrian persons who are participating in this study. Details of selection procedures will be provided later.

## 2 MATERIALS AND METHODS

### 2.1 Ethics approval of the study

This study was conducted at the Department of Oral Medicine of Damascus University, Faculty of Dentistry. The guidelines of the Helsinki Declaration were followed, and Ethics Review Board at Dental school approved the study (ref. no.3962/E dated 30/9/2018). Before participation subjects signed an informed consent form and were informed that they could refrain from the study at any time without any consequences.

### 2.2 Participants, study design, and setting

One hundred persons participated in this present study. All participants were selected from our patients who were referred for general dental treatment in the dental school at Damascus University and they were asked to join this study voluntarily. They were divided into two groups depending on the bruxism status: bruxer individuals group (consisting of 58 females and 18 males) and non‐bruxer group (including 8 females and 16 males) within the range age of 18–40 years (average age: 27 years). Both groups were referred to the laboratory for 25(OH)D levels analysis tests. The bruxism status of all participants was examined according to the recommendations of Pintado et al. (1997) (Table 1), and a 25‐item questionnaire (Bruxism severity classification questionnaire) (Molina et al., 2013) was answered by bruxer patients to determine the severity levels of bruxism (mild, moderate, severe, and extreme severe) 3–5, 6–10, 11–15, 16–25 signs and symptoms, respectively, side by side to the clinical examination (Table 2). All examinations were performed in advance in the Oral Medicine department between 10:00 a.m. and 1:00 p.m. from the beginning of March until the end of November 2019.

**Table 1:** The bruxism status according to recommendations of “Pintado et al.”

	Answer
1. Has anyone heard you grinding your teeth at night?	Yes/No
2. Is your jaw ever fatigued or sore on awakening in the morning?	Yes/No
3. Are your teeth or gums ever sore on awakening in the morning?	Yes/No
4. Do you ever experience temporal headaches on awakening in the morning?	Yes/No
5. Are you ever aware of grinding your teeth during the day?	Yes/No
6. Are you ever aware of clenching your teeth during the day?	Yes/No

*Note*: Subjects who were classified as bruxers gave a positive response to at least two of the six items.

**Table 2:** Bruxism severity classification questionnaire

1. Patient's report of catching himself/herself clenching the teeth during the day;
2. A report of masseter muscle fatigue during the day;
3. Patient's report of masseter muscle fatigue on awakening in the morning;
4. Masseter muscle tension during the day;
5. Masseter muscle tension on awakening in the morning;
6. Patient's report of catching himself/herself grinding or clenching at night;
7. A feeling of jaw locking on awakening at night;
8. Tooth wear, specifically on the anterior and lower teeth on visual inspection;
9. Patient's report of awakening with facial, headache and or TMJ pain in the morning;
10. Patient's report of awakening in the morning with a feeling of pain/discomfort in the teeth;
11. A report of awakening in the morning with a feeling of having slept with the jaws locked, clenching;
12. Toothache on awakening in the morning, not related to caries or a dental lesion;
13. Tooth sensitivity to cold;
14. Hypertrophy of the masseter muscle;
15. Recent history of fracturing teeth and/or restorations;
16. Friends/relatives' report of clenching or grinding the teeth at night;
17. Cervical pain on awakening in the morning;
18. A feeling of body fatigue on awakening in the morning;
19. Patient's report of feeling tired or with a feeling of daytime sleepiness;
20. Presence of cheek biting;
21. Presence of tongue biting or tongue indentations;
22. Exostosis in the maxilla and or in the mandible;
23. Tori in the maxilla and or mandible;
24. Difficulties to open the mouth on awakening in the morning;
25. History of fracture, breaking or excessive damage of an occlusal splint.

*Note*: Mild, moderate, severe, and extreme severe bruxers were those presenting 3–5, 6–10, 11–15, 16–25 signs and symptoms, respectively.

The exclusion criteria for both groups were (Veiga et al., 2015): medication that could interfere with vitamin D metabolism (Demjaha et al., 2019), presence of fixed or removable prosthodontics for more than four units (Kato et al., 2003), presence of severe malocclusion (Lavigne et al., 2007), excessive smoking (Herrero Babiloni & Lavigne, 2018), and myofascial pain dysfunction. Exclusion criteria were assessed in each subject by taking a medical history and additional clinical tests, if needed.

It should be pointed out here that (Bruxism Severity Classification Questionnaire) contains questions related to both awake and sleep bruxism depending on clinical features and examination but it cannot be definitive. Bruxism definitive diagnosis should be supported by some instrumental approaches (polysomnography for sleep bruxism and electromyography for awake bruxism) side by side to clinical examination and self‐reports (Lobbezoo et al., 2013). In our study, we did not determine the type of bruxism (sleep or awake) but we only used the questionnaire to measure the severity of bruxism after confirmation or denial.

Moreover, the limitation of the study sample was due to difficulties finding out volunteers and the limited available budget so that the number of sub‐groups of bruxism severity levels was low.

### 2.3 Laboratory procedure

In the laboratory, blood samples were taken from each patient's arm (the cephalic vein). 25(OH)D levels were analyzed with Roche Total 25‐OH vitamin D kits (based on electro‐chemiluminescence binding assay). According to the expected values of Elecsys® 25‐hydroxyvitamin D test characteristics, the normal range for 25(OH)D concentration in nanogram: <20 ng/ml was considered as deficient, between 21 and 29 ng/ml was considered as insufficient, between 30 and 150 ng/ml was considered as sufficient and finally the concentration of 25(OH)D is more than 150 ng/ml, which was considered as toxicity.

### 2.4 Statistical analysis

The results were analyzed with SPSS (Statistical Package for the Social Science) and *p* values <.05 were considered statistically significant. Spearman's correlation coefficient analysis was used for the statistical analysis.

## 3 RESULTS

Table 3 shows all the collected results for the investigated samples according to the questionnaire (ages were between 18 and 40 years and the maximum gender was females (66 and 34 for males). The laboratory and clinical results are shown in Tables 4, 5, 6.

**Table 3:** All collected details for the study samples

Patient number	Gender, M/F	Age	Non‐bruxer	Bruxer				25(OH)D (ng/ml) (±1.7 ng/ml)		
				Extreme	Severe	Moderate	Mild	Sufficient	Insufficient	Deficiency
1	F	25			×			110		
2	F	21					×	32.2		
3	M	23				×		30.8		
4	M	25	/							17.9
5	F	23				×				19.6
6	M	22	/						24.5	
7	F	24	/					95.9		
8	F	24			×			36.8		
9	F	26	/					33.9		
10	F	27	/					35.8		
11	F	22		×				60.6		
12	F	22			×				22.5	
13	F	22	/						25.1	
14	F	21			×				21.8	
15	F	23			×					20.2
16	F	22	/					50.34		
17	F	23		×					27	
18	F	21		×					24.6	
19	F	21			×			35.63		
20	F	19		×					21	
21	F	22		×				42		
22	F	23				×			25.2	
23	F	22				×				20
24	M	22				×		85		
25	F	23				×		34.88		
26	F	32			×			33.11		
27	F	27			×					16.96
28	F	30				×			29.91	
29	F	30	/						22.05	
30	F	33				×				13.71
31	F	23			×			32.13		
32	F	24			×					10.5
33	F	23				×				12.4
34	F	26				×				10.78
35	F	23				×				12.81
36	M	29	/						29.7	
37	M	25	/						22.23	
38	M	27	/						26.56	
39	M	25	/							16.51
40	F	25				×			24	
41	F	24		x						10.29
42	M	34	/							20.16
43	F	24		x						11.18
44	F	23				×			22.02	
45	M	25				×		31.37		
46	M	21	/					37.63		
47	M	23	/						24.76	
48	M	25	/							19.92
49	M	30	/					40.98		
50	M	37			×			58.84		
51	F	23			×					15.2
52	M	38	/					38.05		
53	M	30	/					46.14		
54	M	20	/						23.6	
55	F	33			×					12.9
56	M	29			×				22	
57	M	32	/						29.44	
58	M	25					×	46.15		
59	M	35					×		25.14	
60	M	39					×		22.85	
61	M	26					×		27.69	
62	F	29	/					49.19		
63	F	23			×					13
64	M	23					×		21.53	
65	F	20				×				20.14
66	F	31			×					14.8
67	F	33		×					25.2	
68	F	27				×				19.3
69	F	27			×					13.3
70	M	37				×		51.22		
71	M	25					×			17.85
72	M	40					×			15.8
73	M	36	/						26.7	
74	F	22			×					13
75	F	39				×			21	
76	F	33		×						15.6
77	M	31				×		97.3		
78	F	22				×				18.98
79	F	20			×					18.2
80	F	33			×					17
81	F	38				×				16.3
82	F	30				×				14.5
83	F	31				×				19.3
84	F	23			×					16.8
85	F	24			×				26.61	
86	F	26				×				17
87	F	26				×			22.2	
88	F	29		×				89		
89	F	21				×				16.3
90	F	23				×				14.7
91	F	29			×					16.8
92	F	24			×			31.95		
93	M	39					×		21.1	
94	M	36				×				18
95	F	29			×					15.5
96	F	29		×						14.2
97	M	27				×		39.49		
98	F	28				×				16.8
99	M	38					×			19.1
100	F	38				×				17

**Table 4:** Shows the relationship between 25(OH)D concentration in the serum for the investigated categories

Total	Bruxism					25(OH)D
	Extreme severe	Severe	Moderate	Mild	Non‐bruxer	
28 (28.0%)	3 (27.2%)	6 (25.0%)	7 (22.5%)	2 (20.0%)	10 (41.7%)	Sufficient
29 (29.0%)	4 (36.4%)	4 (16.7%)	6 (19.4%)	5 (50.0%)	10 (41.7%)	Insufficient
43 (43.0%)	4 (36.4%)	14 (58.3%)	18 (58.1%)	3 (30.0%)	4 (16.6%)	Deficient
100 (100.0%)	11 (11.0%)	24 (24.0%)	31 (31.0%)	10 (10.0%)	24 (24.0%)	Total

Abbreviation: 25(OH)D, 25‐hydroxyvitamin D.

**Table 5:** Exhibits the descriptive statistical analysis for 25(OH)D levels in connection with bruxism

Mean accuracy (95%)		25(OH)D				*n*	Bruxism
Maximum	Minimum	Highest value	Lower value	SD	Mean		
39.85	25.90	95.90	16.51	16.52	32.88	24	Non‐bruxer
31.29	18.59	46.15	15.80	8.87	24.94	10	Mild
33.32	19.06	97.30	10.78	19.44	26.19	31	Moderate
34.44	16.69	110.00	10.50	21.02	25.57	24	Severe
47.28	14.66	89.00	10.29	24.28	30.97	11	Extreme severe

Abbreviation: 25(OH)D, 25‐hydroxyvitamin D.

**Table 6:** *P* values for Spearman's correlation coefficient test

*r*	Assignment	Relationship	Bruxism			25(OH)D
			*p* value	*n*	Correlation coefficient	
Weak	Positive	Yes	.039	100	0.207	Measured values
Weak	Positive	Yes	.007	100	0.268	Classifications

Abbreviation: 25(OH)D, 25‐hydroxyvitamin D.

Table 4 shows the relationship between 25(OH)D concentrations in the serum for the investigated categories.

In the non‐bruxer group, both sufficient and insufficient 25(OH)D levels were equally distributed (41%) while deficiency was spotted for about (16%) of the group.

These ratios had been altered in the mild‐bruxer group to become 50%, 30%, and 20% for insufficient, deficient, and sufficient 25(OH)D, respectively. Almost, the average of 58% of the patients in both moderate and severe bruxer groups have suffered from 25(OH)D deficiency. Finally, the extremely severe bruxer group was of about 36% of 25(OH)D levels were noticed in the study samples. Generally speaking, bruxism activity has been associated with a deficiency of 25(OH)D as observed in our recorded measurements. The *p* value showed a fundamental relationship between the levels of bruxism and in comparison with numerical values of 25(OH)D measured concentration. Table 5 exhibits the descriptive statistical analysis for 25(OH)D levels in connection with bruxism. Table 6 shows the *p* values for Spearman's correlation coefficient test.

## 4 DISCUSSION

Vitamin D is a prohormone that has a key role in calcium and phosphate balance and bone structure in the body (Autier et al., 2014). Sufficient vitamin D supply can be concluded through dietary vitamin D intake from many resources, mainly through skin exposure to solar ultraviolet (UV) B radiation or from different nutrition resources (Zittermann, 2017).

In addition, it is important to highlight that the levels of vitamin D synthesis after sunlight exposure are strongly influenced by many different factors, including skin pigmentation, use of sunscreen, latitude, season, and age. On the other hand, the contribution of diet intake and supplementation to serum 25(OH)D levels appear nonlinear behavior and can also be influenced by several factors, including baseline levels of 25(OH)D, body mass index and percentage of body fat, age, and calcium intake, among others (Mazahery & Von Hurst, 2015).

It should be noted that the above reasons may interfere with the interpretation of the different concentrations of 25(OH)D in the individuals in the investigated samples, but in our study, we measured the current individual's 25(OH)D blood concentrations despite the influencing factors taking into consideration the exclusion guidelines.

Vitamin D deficiency is prevalent throughout the world. It is estimated that over 50% of the world's population has low vitamin D levels (i.e., serum levels of 25(OH)D < 30 ng/ml or 75 nmol/L) (Wimalawansa et al., 2018). Our recorded observation is consistent with the international results standard that the vitamin D deficiency which was about 43% of the total investigated samples reported in our study.

In the past decade, vitamin D has been actually focusing on the keen interest because beyond these known roles data from ecological and observational studies have shown associations between serum 25(OH)D concentrations (usually used as a proxy for an individual's vitamin D status) and psychological status of individuals (Casseb et al., 2019).

Thus majority of the clinical literature that deals with the possible role of vitamin D in psychological disorders established an inverse association between serum 25(OH)D levels and depressive symptoms as well as anxiety disorder (Han et al., 2018; Sherchand et al., 2018). Some studies, however, failed to find significant associations (Michaëlsson et al., 2018; Şahin Can et al., 2017).

In contrast, some researchers propose a hypothesis that sleep disorders have become epidemic because of widespread vitamin D deficiency due to the association between brain regions of sleep‐awake regulation with vitamin D target neurons in the diencephalon, while several brainstem nuclei suggest direct central effects of vitamin D on sleep (Gominak & Stumpf, 2012).

Bruxism is a repeated jaw‐muscle activity characterized by clenching or grinding of the teeth and/or by bracing or thrusting of the mandible. It can occur during sleep (sleep bruxism) or during wakefulness (awake bruxism) (Lobbezoo et al., 2013).

Lobbezoo et al. proposed a bruxism grading system to assign a specific assessment to it, which would lead to valid results. Three diagnostic levels (probable, possible, and definite) were proposed.

The probability is based on self‐reports only and the possibility of bruxism depends on self‐reports and clinical examination. Instrumental approaches (polysomnography for sleep bruxism and electromyography for awake bruxism) are added to definite the diagnosis (Lobbezoo et al., 2013).

Our study relied on determining the possibility of bruxism through self‐reports (questionnaire) and clinical examination only due to the lack of instrumental approaches at Damascus University.

Bruxism etiology is multifactorial; therefore, the psycho‐emotional is considered one of the most important etiological factors (Singh et al., 2015). Recently, Manfredini et al. (2015) also pointed out that the main cause of this parafunction which is leading to a sleep disorder, explained by the arousal.

It should be pointed here that the International Classification of Sleep Disorder (ICSD‐R) states that 85–90% of the general population grind their teeth to a degree at some point during their life, although only 5% will develop a clinical condition (American Academy of Sleep Medicine, 2001). In our study, the results are pointed out that the majority of patients with bruxism are distributed in the medium to severe levels. It should be also noted that the results of very severe dental grinding were determined and seen only by female patients and no extreme severe cases of bruxism were observed in male patients. These results can be explained by the fact that the number of female patients was 66, which is higher than male patients. In addition to that the results of the questionnaire used showed higher values of bruxism levels when there was a case of sleep bruxism (SB) and awake bruxism (AB) together in individuals, as the number of signs and symptoms recorded in the questionnaire increased. The interpretation of this may be related to the (AB) with females is more popular than males, while the (SB) affects both males and females with similar percentage (Macedo et al., 2007; Shetty et al., 2010).

According to the physiopathology of bruxism, several studies have highlighted the existence of a central administration in the mechanism of bruxism episodes. Taking the presence of a neurological mechanism in psychological factors and sleep stability for both bruxism etiology and 25(OH)D concentrations, due to the spread of vitamin D receptors (VDR) in multiple areas of the central nervous system, may lead to obtaining an investigation about the possible role of serum 25‐hydroxyvitamin D levels in reducing or exacerbating bruxism levels.

It is widely accepted that bruxism has multifactorial etiology, and the interferences of these factors in individuals may make it more difficult to measure and identify them specifically (Demjaha et al., 2019).

Despite the individual psychological state study or polysomnography (PSG) studies, the current reported research was conducted to measure the levels of 25(OH)D that has a potential role in the psychological state and the stability of sleep of the individuals, which, on the other hand, has a participation in the occurrence of bruxism. The results showed that there is a relationship between the levels of bruxism and levels of 25(OH)D concentration in the blood.

## 5 CONCLUSION

Within the limitations of the current study, it can be concluded that 25(OH) D concentrations have a possible association with bruxism level variation.

## CONFLICT OF INTERESTS

The authors declare that there are no conflict of interests.

## AUTHOR CONTRIBUTIONS

Bushra A. W. Allaf wrote the manuscript and provided data, filled out all patient self‐reports. Bushra A. W. Allaf conducted the clinical examination, blood samples collection, and conducted all statistical analyses. Mahmoud Abdul‐Hak encouraged Bushra A. W. Allaf to investigate the vitamin D role possibility and supervised the findings of this work. Both authors discussed the results and contributed to the final manuscript.

## Data Availability

The data that support the findings of this study are available from the corresponding author upon reasonable request.

## References

[cre2530-bib-0001] American Academy of Sleep Medicine . (2001). *International classification of sleep disorders, revised: Diagnostic and coding manual* (Vol 22).

[cre2530-bib-0002] Autier, P. , Boniol, M. , Pizot, C. , & Mullie, P. (2014). Vitamin D status and ill health: A systematic review. The Lancet Diabetes & Endocrinology, 2(1), 76–89. 24622671 10.1016/S2213-8587(13)70165-7

[cre2530-bib-0003] Berridge, M. J. (2016). Vitamin D, reactive oxygen species and calcium signalling in ageing and disease. Philosophical Transactions of the Royal Society B: Biological Sciences, 371, 20150434. 10.1098/rstb.2015.0434 PMC4938033 27377727

[cre2530-bib-0004] Casseb, G. A. , Kaster, M. P. , & Rodrigues, A. L. S. (2019). Potential role of vitamin D for the management of depression and anxiety. CNS Drugs, 33(7), 619–637. 31093951 10.1007/s40263-019-00640-4

[cre2530-bib-0005] Chu, M. P. , Alagiakrishnan, K. , & Sadowski, C. (2010). The cure of ageing: vitamin D—Magic or myth? Postgraduate Medical Journal, 86, 608–616. 20971712 10.1136/pgmj.2010.101121

[cre2530-bib-0006] Demjaha, G. , Kapusevska, B. , & Pejkovska‐Shahpaska, B. (2019). Bruxism unconscious oral habit in everyday life. Open Access Macedonian Journal of Medical Sciences, 7(5), 876–881. 30962854 10.3889/oamjms.2019.196 PMC6447347

[cre2530-bib-0007] Gominak, S. C. , & Stumpf, W. (2012). The world epidemic of sleep disorders is linked to vitamin D deficiency. Medical Hypotheses, 79(2), 132–135. 22583560 10.1016/j.mehy.2012.03.031

[cre2530-bib-0008] Han, B. , Zhu, F.‐X. , Yu, H.‐F. , Liu, S. , & Zhou, J.‐L. (2018). Low serum levels of vitamin D are associated with anxiety in children and adolescents with dialysis. Scientific Reports, 8(1), 1–6. 29654252 10.1038/s41598-018-24451-7 PMC5899084

[cre2530-bib-0009] Hassan, W. , Eduardo Barroso Silva, C. , Mohammadzai, I. U. , Batista Teixeira Da Rocha, J. , & Landeira‐Fernandez, J. (2014). Association of oxidative stress to the genesis of anxiety: Implications for possible therapeutic interventions. Current Neuropharmacology, 12, 120–139. 24669207 10.2174/1570159X11666131120232135 PMC3964744

[cre2530-bib-0010] Herrero Babiloni, A. , & Lavigne, G. J. (2018). Sleep bruxism: A “bridge” between dental and sleep medicine. Journal of Clinical Sleep Medicine, 14(8), 1281–1283. 30092910 10.5664/jcsm.7254 PMC6086949

[cre2530-bib-0011] Kato, T. , Montplaisir, J. , Guitard, F. , Sessle, B. , Lund, J. , & Lavigne, G. (2003). Evidence that experimentally induced sleep bruxism is a consequence of transient arousal. Journal of Dental Research, 82(4), 284–288. 12651932 10.1177/154405910308200408

[cre2530-bib-0012] Koundourakis, N. E. , Avgoustinaki, P. D. , Malliaraki, N. , & Margioris, A. N. (2016). Muscular effects of vitamin D in young athletes and non‐athletes and in the elderly. Hormones, 15(4), 471–488. 28222403 10.14310/horm.2002.1705

[cre2530-bib-0013] Lavigne, G. J. , Huynh, N. , Kato, T. , Okura, K. , Adachi, K. , Yao, D. , & Sessle, B. (2007). Genesis of sleep bruxism: Motor and autonomic‐cardiac interactions. Archives of Oral Biology, 52(4), 381–384. 17313939 10.1016/j.archoralbio.2006.11.017

[cre2530-bib-0014] Lobbezoo, F. , Ahlberg, J. , Glaros, A. G. , Kato, T. , Koyano, K. , Lavigne, G. J. , de Leeuw, R. , Manfredini, D. , Svensson, P. , & Winocur, E. (2013). Bruxism defined and graded: An international consensus. Journal of Oral Rehabilitation, 40(1), 2–4. 23121262 10.1111/joor.12011

[cre2530-bib-0015] Macedo, C. R. , Silva, A. B. , Machado, M. A. C. , Saconato, H. , & Prado, G. F. (2007). Occlusal splints for treating sleep bruxism (tooth grinding). Cochrane Database of Systematic Reviews, ​(4), 005514. 10.1002/14651858.CD005514.pub2 PMC8890597 17943862

[cre2530-bib-0016] Manfredini, D. , Ahlberg, J. , Winocur, E. , & Lobbezoo, F. (2015). Management of sleep bruxism in adults: A qualitative systematic literature review. Journal of Oral Rehabilitation, 42(11), 862–874. 26095208 10.1111/joor.12322

[cre2530-bib-0017] Mazahery, H. , & Von Hurst, P. R. (2015). Factors affecting 25‐hydroxyvitamin D concentration in response to vitamin D supplementation. Nutrients, 7(7), 5111–5142. 26121531 10.3390/nu7075111 PMC4516990

[cre2530-bib-0018] Michaëlsson, K. , Melhus, H. , & Larsson, S. C. (2018). Serum 25‐hydroxyvitamin D concentrations and major depression: A Mendelian randomization study. Nutrients, 10(12), 1987. 10.3390/nu10121987 PMC6316636 30558284

[cre2530-bib-0019] Molina, O. F. , Santos, Z. C. , Simião, B. R. H. , Marchezan, R. F. , de Paula, N. , & Gama, K. R. (2013). A comprehensive method to classify subgroups of bruxers in temporomandibular disorders (TMDs) individuals: Frequency, clinical and psychological implications. RSBO Revista Sul‐Brasileira de Odontologia, 10(1), 11–19.

[cre2530-bib-0020] Moon, R. J. , Curtis, E. M. , Davies, J. H. , Cooper, C. , & Harvey, N. C. (2017). Seasonal variation in Internet searches for vitamin D. Archives of Osteoporosis, 12(1), 28. 28283938 10.1007/s11657-017-0322-7 PMC5346128

[cre2530-bib-0021] Pintado, M. R. , Anderson, G. C. , DeLong, R. , & Douglas, W. H. (1997). Variation in tooth wear in young adults over a two‐year period. The Journal of Prosthetic Dentistry, 77(3), 313–320. 9069087 10.1016/s0022-3913(97)70189-6

[cre2530-bib-0022] Şahin Can, M. , Baykan, H. , Baykan, Ö. , Erensoy, N. , & Karlıdere, T. (2017). Vitamin D levels and vitamin D receptor gene polymorphism in major depression. Psychiatria Danubina, 29(2), 179–185. 10.24869/psyd.2017.179 28636576

[cre2530-bib-0023] Santos, P. , Herrmann, A. P. , Elisabetsky, E. , & Piato, A. (2019). Anxiolytic properties of compounds that counteract oxidative stress, neuroinflammation, and glutamatergic dysfunction: A review. Brazilian Journal of Psychiatry, 41, 168–178. 30328963 10.1590/1516-4446-2018-0005 PMC6781690

[cre2530-bib-0024] Sherchand, O. , Sapkota, N. , Chaudhari, R. K. , Khan, S. A. , Baranwal, J. K. , Pokhrel, T. , Das, B. , & Lamsal, M. (2018). Association between vitamin D deficiency and depression in Nepalese population. Psychiatry Research, 267, 266–271. 29940458 10.1016/j.psychres.2018.06.018

[cre2530-bib-0025] Shetty, S. , Pitti, V. , Babu, C. S. , Kumar, G. S. , & Deepthi, B. (2010). Bruxism: A literature review. The Journal of Indian Prosthodontic Society, 10(3), 141–148. 21886404 10.1007/s13191-011-0041-5 PMC3081266

[cre2530-bib-0026] Singh, P. K. , Alvi, H. A. , Singh, B. P. , Singh, R. D. , Kant, S. , Jurel, S. , Singh, K. , Arya, D. , & Dubey, A. (2015). Evaluation of various treatment modalities in sleep bruxism. The Journal of Prosthetic Dentistry, 114(3), 426–431. 26004173 10.1016/j.prosdent.2015.02.025

[cre2530-bib-0027] Veiga, N. , Ângelo, T. , Ribeiro, O. , & Baptista, A. (2015). Bruxism–literature review. International Journal of Dentistry and Oral Health, 1(5), 1–5.

[cre2530-bib-0028] Wimalawansa, S. J. , Razzaque, M. S. , & Al‐Daghri, N. M. (2018). Calcium and vitamin D in human health: Hype or real? The Journal of Steroid Biochemistry and Molecular Biology, 180, 4–14. 29258769 10.1016/j.jsbmb.2017.12.009

[cre2530-bib-0029] Zittermann, A. (2017). The biphasic effect of vitamin D on the musculoskeletal and cardiovascular system. International Journal of Endocrinology, 2017, 1–11. 10.1155/2017/3206240 PMC5587949 28912809

